# Evaluating a general pediatric/adult genetic counseling clinic in a Midwest medical center

**DOI:** 10.1002/jgc4.1603

**Published:** 2022-07-04

**Authors:** Charlie King, Shelly Nielsen, Andrew Schmanski, Omar Abdul‐Rahman, Kristen P. Fishler

**Affiliations:** ^1^ Nebraska Hematology‐Oncology P.C. Lincoln Nebraska USA; ^2^ University of Nebraska Medical Center, Munroe‐Meyer Institute for Genetics & Rehabilitation Omaha Nebraska USA

**Keywords:** autonomous counseling, genetic counseling, genetic counseling clinic, genetic counselor, increased access to care, service delivery models

## Abstract

In 2018, the Munroe‐Meyer Institute for Genetics & Rehabilitation (MMI) at the University of Nebraska Medical Center (UNMC) in Omaha, NE created a genetic counseling clinic (GCC) to increase access to genetics services and decrease the time spent between a referral and being seen in a general genetics outpatient clinic. In the GCC, genetic counselors led patient encounters and geneticists served as advisors, rather than primary providers. We conducted a chart review of 109 patients seen in the GCC from November 1, 2018, to March 16, 2020, and obtained information regarding patient demographics, indications, and clinical recommendations as a result of the visit. Most patients seen in this clinic were female (65.1%) and aged 19 years of age or older (54.1%). The primary indications for patients in this clinic included review genetic test results (42.2%), coordination of genetic testing for a known familial variant (30.2%), and concerns for personal or family history suspicious of a genetic condition without dysmorphic features (24.8%). The average patient wait time between referral date and appointment date in the GCC was 49.8 days. The two most common clinical recommendations made by genetic counselors in the GCC were genetic testing (56.1%) and/or follow‐up with specialist (26.5%). These specialists primarily included endocrinology (*n* = 5), neurology (*n* = 4), cardiology (*n* = 4), ophthalmology (*n* = 3), and audiology (*n* = 3). We found that the GCC model may be appropriate for patients with (1) genetic test results requiring interpretation, (2) a known familial variant or (3) genetic testing recommended by a specialist physician. Descriptions of the indications and recommendations for patients seen in this GCC provide a framework for potential implementation of a GCC in other regions across the nation.

What is known about this topic

Utilizing a general genetic counseling clinic can expand patient access to genetic services by complementing traditional clinic models and utilizing the strengths of the genetic counselor healthcare provider.

What this paper adds to the topic

Implementing a genetic counseling clinic, where genetic counselors practice with minimal supervision of a geneticist, increased access to genetic counseling. For patients seen in the GCC, there was a shorter wait time between the date of referral and date of the visit when compared to a general genetics clinic in the same region. Indications seen in this clinic include patients with genetic test results requiring interpretation, consent for genetic testing to identify previously discovered familial variant(s), or genetic testing recommended by a specialist physician based on personal and/or family history.

## 1 BACKGROUND

In general genetics clinics, geneticists and genetic counselors work together to provide care. Prior studies have identified that patient access to in‐person general genetics care was mainly limited by wait time and travel distance (Cohen et al., 2016; Greenberg et al., 2020). A recent study identified that 62% of medical geneticists were practicing in a clinic with a patient wait time of greater than 1 month (Maiese et al., 2019). It is known that a genetic diagnosis can lead to tailored management recommendations, school and government agency supports, and specialist referrals. Early diagnosis, treatment, and support allow patients to maximally benefit from referrals and services. Conversely, delay in proper diagnosis and referrals has been shown to cause adverse outcomes (Martínez‐Molina et al., 2020; Murni et al., 2021; Zurynski et al., 2017).

While the number of practicing genetic counselors has grown over the last decade through the addition of many genetic counseling training programs, the number of practicing medical geneticists has not seen the same growth (Bacino et al., 2020; Hoskovec et al., 2018). Training to become a medical geneticist in the United States traditionally requires 4 years of undergraduate study, 4 years of medical school, 2–3 years of primary residency training, and 2 years of genetics medicine and genomics fellowship training. This equates to 12 years of training in total, and up to 9 years of training following an undergraduate‐level education. In comparison, training to become a genetic counselor in the United States and Canada requires 4 years of undergraduate study, followed by a two‐year master's degree from an accredited genetic counseling program. Additionally, there has been a stark difference in the ability to fill available training positions between genetic counselors and clinical geneticists. Genetic counseling programs typically have many applicants, and the number of trainees is primarily limited by the number of available supervision sites rather than applicants interested in filling positions (Berg et al., 2018). In contrast, approximately one‐third of available genetics residency positions remain unfilled (Jenkins et al., 2021). These differences in training requirements and ability to fill training positions influence each profession's ability to respond to the growing demand for clinical genetics services.

Alternative approaches to the traditional model of clinical genetics service delivery, such as genetic counseling clinics (GCCs), are needed as patient wait times and genetic provider caseloads rise (Maiese et al., 2019). In these GCCs, genetic counselors lead patient encounters, make genetic testing recommendations, and recommend appropriate specialist follow‐up. The majority of the time in a GCC session may be spent discussing the benefits, limitations, logistics, and implications of genetic testing, or providing post‐test genetic counseling; pediatric visits in a traditional model also include a detailed physical examination and discussion of these physical findings. In many cases, genetic counselors provide genetics services without the need for additional genetics follow‐up in an outpatient setting. One example includes the GCC at Vanderbilt, where 80% of patients first seen in their GCC did not need additional appointments with a clinical geneticist (Hannig et al., 2014).

The Munroe‐Meyer Institute for Genetics & Rehabilitation (MMI) at the University of Nebraska Medical Center (UNMC) implemented a GCC in 2018—following the addition of several genetic counselors—to expand access to clinical genetics services. UNMC primarily operates in Omaha, Nebraska, a midwestern city with a population of nearly 500,000. In addition to providing care to the residents of Omaha, UNMC provides care to many suburban and rural patients throughout Nebraska and eastern Iowa.

The goal of this study was to describe the patient population, reason for referral, wait time between referral and visit date, and clinical recommendations for patients seen in this clinic from November 1, 2018, to March 16, 2020. We predicted coordination of genetic testing for a familial variant would be the primary indication for being seen in the UNMC GCC. Additionally, we predicted that most patients would not require follow‐up in a general genetics clinic and that patient wait times from time of referral to visit date would be reduced compared with wait times for a general genetics outpatient visit in the same region.

## 2 METHODS

We conducted a retrospective chart review of patients seen in the UNMC GCC from November 1, 2018, to March 16, 2020. This study was approved by the UNMC Institutional Review Board (IRB #: 452‐20‐EP). All data were de‐identified, and adequate safeguards were established to protect subjects involved in the study.

### 2.1 Participants

We accessed 109 patient records with appointment type 'GCCONSULT' in the UNMC Epic electronic medical record database from November 1, 2018, to March 16, 2020. Although the GCC was operating for 9 months prior to November 2018, visits were not listed in the medical record system with a universal label prior to November 2018, which limited our ability to review data associated with these types of encounters. During the study period, all patients were seen in person.

All patient referrals were evaluated by the patient information office and genetic counselors (when needed) to determine eligibility for the GCC. The original date of referral was not available for most adult patient encounters. To supplement referral dates noted in Epic, paper referral records were also reviewed. Following cross‐examination of referrals noted in Epic with these paper files, the amount of time between date of appointment and date of referral was measured for 32 patient visits. A majority of GCC pediatric visits (22/30) had referral data available; therefore, GCC pediatric wait time from referral to visit date was reported for comparison to a general genetics clinic in the same region.

### 2.2 Procedures

We automatically extracted the following variables from the electronic medical record: patient zip code, patient sex, patient age, referral indication, wait time between date of referral and date of appointment, and insurance provider. One member of the research team manually extracted the following variables from the electronic medical record: types of genetic test results reviewed, clinical recommendations, mode of referral, and visit type.

During data extraction, information was coded into several unique categories. Mode of referral was coded into one of four categories: internal genetics referral, self‐referral, primary care provider, and specialist referral. Visit type was coded into one of four categories: pediatric, prenatal, preconception, and adult. Patients who were 19 or older were classified as an adult, and patients 18 or younger were coded as a pediatric patient. Patients who were pregnant at the time of the visit were coded as prenatal patients. All patients who were noted to be pregnant at the time of the visit discussed topics related to the health of their fetus. Patients who were not pregnant and were primarily seen to discuss carrier screening and fertility implications of carrier screening were coded as preconception patients. Referral indications were coded into one of the five categories: review genetic test results, genetic testing for a familial variant, family history suspecting genetic disease, personal history suspecting genetic disease, and coordination of carrier screening. In a previous study reviewing medical management adherence in a genetic counseling setting, clinical recommendations were grouped into three categories: follow‐up with genetics, referral to non‐genetics specialist, and testing (genetic testing, imaging, and other blood, urine, or sweat tests) (Rutherford et al., 2014). These categories served as a model for how we categorized clinical recommendations in this review and were coded into one of five categories: follow‐up with genetics, follow‐up with specialist, familial genetic testing, genetic testing for a family member, and novel genetic testing. Follow‐up with specialist included any recommendation for the patient to see a healthcare provider who was not a board‐certified geneticist or genetic counselor. Familial genetic testing was defined as genetic testing for a specific mutation that had been previously identified in their family. Genetic testing for family was defined as a recommendation for other family members to be referred for genetic testing. Novel genetic testing was defined as genetic testing that was recommended for patients without a known variant in the family.

Mode of referral was not completely documented in all patient charts in the GCC. For instances in which the date and referring provider were not listed, we listed the primary care provider on file as the referring provider. To mitigate this limitation and improve data accuracy, we asked genetic counselors working in the clinic to cross‐reference the referral records to obtain mode of referral to supplement the primary data extraction.

### 2.3 Data analysis

Descriptive statistics including mean, median, and range were used to analyze patient sex, visit type, mode of referral, referral indication, clinical recommendation(s), specialist type, and wait time.

## 3 RESULTS

### 3.1 Patient demographics

Table 1 summarizes the demographics of patients seen in the GCC.

**TABLE 1:** Patient demographic

	Patients (*N* = 109)
Sex	*n* (%)
Female	71 (65.1)
Male	38 (34.9)
Visit Type	
Adult	46 (42.2)
Pediatric	33 (30.2)
Preconception	15 (13.8)
Prenatal	1 (0.92)

Age (years)	Average	Median	Range
All Visits	31.2	31	0–77
Adult	43.5	39.5	22–77
Pediatric	6.23	5.5	0–17
Preconception	31.3	31	25–38
Prenatal	32	32	32

### 3.2 Referral information

Table 2 summarizes mode of referral and referral indications for the GCC. Patients seen in this clinic were classified as having only one referral indication per visit type. The most common referral indication for this clinic was to review genetic test results, which involved interpretation and explanation of their genetic test results, as well as clearly outlining appropriate medical surveillance recommendations. Genetic tests reviewed with patients included microarray, whole‐exome sequencing, gene panel testing, carrier screening, single variant testing, and karyotype. Patients who would not clinically benefit from a detailed physical examination were also referred to the GCC based on a personal or family history of genetic disease including long QT syndrome, thalassemia, Stargardt disease, cerebellar ataxia, spontaneous coronary artery dissection, hemochromatosis, and neurofibromatosis type 2.

**TABLE 2:** Referral characteristics

	Patients (*N* = 109)
**Mode of referral**	*n* (%)
Primary care provider	53 (48.6)
Specialist referral	23 (21.1)
Internal genetics referral	21 (19.3)
Self‐referral	12 (11.0)
**Referral indication**	
Review genetic test results	46 (42.2)
Genetic testing for a familial variant	33 (30.2)
Family history suspecting genetic disease	15 (13.8)
Personal history suspecting genetic disease	12 (11.0)
Coordinate carrier screening	3 (2.75)

### 3.3 GCC wait time

The average patient wait time between referral date and appointment date in the GCC was 49.8 days. This referral information was available for 32 out of 109 total visits in the GCC. Twenty‐two of the 32 visits with available referral data were for pediatric indications. The mean wait time between referral and GCC appointment date for these pediatric patients (*n* = 22) was 51.3 days. In comparison, the average wait time for the general genetics clinic at Children's Hospital & Medical Center, Omaha (CHMC) ranged between 6 and 9 months throughout the timeframe of the chart review and was most recently reported to be 182 days (Amy Iske, Central Scheduler at CHMC, personal communication, May 2022; Mayra Marquez, Central Scheduler at CHMC, personal communication, February 2021).

### 3.4 Clinical recommendations

Genetic counselors provided clinical recommendations for 82 of the 109 GCC encounters. In some cases, more than one clinical recommendation was made in an encounter leading to a total of 98 clinical recommendations. Table 3 summarizes the 98 clinical recommendations made for 82 patient encounters in the GCC. Many patients (55%) seen in this clinic received a recommendation for either novel genetic testing or familial genetic testing. The most frequent recommendation made in this clinic was familial genetic testing (33.7%). Other follow‐up recommendations included follow‐up with a specialist (26.5%), novel genetic testing (22.4%), follow‐up with genetics (12.2%), and genetic testing for a family member (5.1%).

**TABLE 3:** Clinical and specialist recommendations

**Clinical recommendation**	*N* = 98 (%)
Familial genetic testing	33 (33.7)
Follow‐up with specialist	26 (26.5)
Novel genetic testing	22 (22.4)
Follow‐up with genetics	12 (12.2)
Genetic testing for family	5 (5.1)
**Specialist recommendation**	*N* = 31 (%)
Endocrinology	5 (16.1)
Neurology	4 (12.9)
Cardiology	4 (12.9)
Ophthalmology	3 (9.7)
Audiology	3 (9.7)
Huntington center for excellence	3 (9.7)
Other (Developmental, Urology, Immunology, ENT)	9 (29.0)

Follow‐up with specialist recommendations was further examined. A total of 31 specialist recommendations were made for 26 individual patients. Specialist recommendations were diverse; no specialty received more than 20% of the follow‐up recommendations. Endocrinology was the most common specialist recommendation (*n* = 5). Other specialists commonly recommended to patients included neurology (*n* = 4), cardiology (*n* = 4), ophthalmology (*n* = 3), and audiology (*n* = 3).

## 4 DISCUSSION

This study analyzed patient demographics, referral characteristics, wait time from referral to visit date, and clinical and specialist recommendations for 109 patients seen in the GCC. Common indications for patients seen in this GCC included review of genetic test results, genetic testing for a familial variant, or patients without dysmorphic features with a personal/family history suspicious of a genetic condition. Our study identified that the GCC had a shorter wait time from referral to visit date compared with a general pediatric genetics wait time in the same region. Visits in the GCC led to a variety of specialist follow‐up recommendations for both adult and pediatric visit types. Most patients seen in the GCC did not require subsequent follow‐up in a traditional outpatient general genetics clinic, thereby decreasing the number of patients added to the genetics wait list.

### 4.1 Common indications for the GCC

Most patients seen in the GCC were adult and pediatric patients who previously had genetic testing. While reviewing these genetic testing results, genetic counselors further discussed the implications of results, provided support resources, and triaged to additional follow‐up services.

A second indication for this clinic was to consent for genetic testing to identify whether a patient had a previously discovered familial variant. In addition to coordination of testing for appropriate variant(s), genetic counselors demonstrated the ability to address ethical issues related to testing in the GCC. An example of this involved a case where two pediatric patients were referred to the GCC for *HFE* genetic testing based on a family history of hemochromatosis, an adult‐onset condition. Offering pediatric genetic testing for adult‐onset conditions presents ethical, social, and legal challenges. Pediatric medical management recommendations for adult‐onset conditions are not currently well‐established (Anderson et al., 2015). Because of this, many experts recommend deferring genetic testing for adult‐onset conditions until children reach the age of consent (Fenwick et al., 2017). Genetic counselors understand the medical and psychosocial impacts of genetic testing and are trained to address these nuances with children and their parents to identify whether genetic testing should be ordered. For these two patients, genetic counseling prompted the family to defer genetic testing until the child was older to protect their autonomy.

Individuals with conditions that require a physical examination by a geneticist are typically referred to a general genetics clinic. However, some patients may receive genetic testing recommendations from another specialist when clinically indicated by the personal or family history, without ever seeing a geneticist. Therefore, it is not surprising that a third indication for the GCC was to coordinate genetic testing after a patient with a personal/family history suspicious of a genetic condition without dysmorphic features had been evaluated by another specialist. Genetic counselors ordered genetic testing for conditions including long QT syndrome, thalassemia, Stargardt disease, cerebellar ataxia, spontaneous coronary artery dissection, hemochromatosis, and neurofibromatosis type 2, based on a prior specialist's evaluation. Common across all of these conditions was that clinical phenotype information came from non‐geneticist providers such as ophthalmologists, neurologists, cardiologists, gastroenterologists, and otolaryngologists. The GCC creates the opportunity for patients who do not need a physical examination by a geneticist to be seen in a timely manner.

### 4.2 Increasing access to genetic services

When comparing the average wait times of the pediatric visit types in the GCC to the general genetics clinic at CHMC in Omaha, NE, the GCC wait time was shorter by an average of 130.7 days. This comparison was made using a 6 month wait time for the general genetics clinic at CHMC and can be considered conservative given the reported wait time range of 6–9 months to be scheduled in the general genetics clinic at CHMC during the timeframe of the chart review (Amy Iske, Central Scheduler at CHMC, personal communication, May 2022). The CHMC genetics clinic wait time is consistent with trends seen in the literature. In one nationwide study, 30% of geneticists had new patient wait times that were greater than 90 days (Maiese et al., 2019). Thus, this study demonstrates the GCC's ability to provide more timely genetics services than a general genetics clinic within the same hospital network, while minimizing further additions to the general genetics waitlist. Patients, referring providers, and genetics providers can all benefit from reduced patient wait times. Timely genetic counseling services may also minimize the stress and anxiety commonly seen in parents of children who receive a positive test result from genetic testing (Dinc & Terzioglu, 2006). The development of a GCC within a health system is one strategy to increase the accessibility of genetic experts and address the concern for appropriate result interpretation.

As the application of genetic testing continues to expand, experienced genetic professionals have expressed concern for improper analysis, interpretation, and result follow‐up by other non‐genetics specialists (Korngiebel et al., 2016). From provider perspectives, neglecting to appropriately follow‐up with genetic testing may be classified as genomic malpractice which can result in liability claims (Marchant et al., 2020). These risks may be highest for non‐genetic providers.

In the UNMC GCC, certified genetic counselors who are licensed in the state of Nebraska are the primary providers seeing patients on a weekly basis. Clinical geneticists are also present in the GCC to help with case management, answer questions that are outside the scope of a genetic counselor's expertise, and to sign off and bill for the appointment. Examples of questions that fall outside of the genetic counselor's scope in the GCC may include questions about symptom management or other medical questions unrelated to the primary indications for the visit. Currently, many insurance companies, including the U.S. Centers for Medicare and Medicaid Services and commercial insurance payers, lack consistent genetic counseling service coverage (Spinosi et al., 2021). Future updates to Medicare law and billing procedures could alter whether clinical geneticists need to be present during these visits as well as the billing practices of genetic counselors who provide care in the GCC.

**FIGURE 1:**
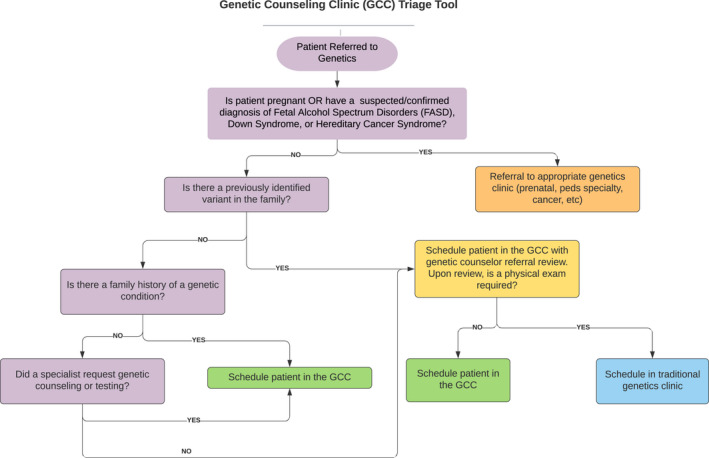
Nebraska medicine genetic counseling clinic (GCC) triage tool

Increasing access to genetic testing and/or genetic counseling to meet current and future demand for genetic services is limited by various factors including the number of people entering the workforce. (*Information on Genetic Counselor and Medical Geneticist Workforces, Report to Congressional Committees* 2020). Implementing alternative service delivery models is one approach to increase access to genetic services. There has been interested within the genetic counseling community, specifically within the National Society of Genetic Counselors Pediatric Special Interest Group discussion board, to develop triage and/or workflows to support genetic counseling clinics across the country (Jacober, 2021). Based on the findings from this study and engagement with the UNMC scheduling staff, we created a triage chart to meet this need and assist other genetic counselors in creating these types of clinics (Figure 1).

### 4.3 Potential downstream revenue from the GCC

Most patients seen in the GCC did not need to follow‐up with genetics and were instead recommended follow‐up with other specialists. The GCC team made recommendations for follow‐up to 10 different specialist types. It is known in other specialty settings, such as cancer genetic counseling, that GC visits can create downstream revenue by appropriately recommending patients have additional specialist visits and screenings. One such example in this setting includes patients with an identified pathogenic variant generating an estimated average of $77,000 for a healthcare institution following an appointment with a genetic counselor (Mauer et al., 2021). For the pediatric visit type, a genetic diagnosis may inform similar and potentially longer‐term evaluations over the course of their life. Our study identifies the potential to impact downstream revenue within the local Nebraska Medicine Healthcare system. This study found that 87.8% of patients did not need to follow‐up with a clinical geneticist after their appointment, which was comparable to a Vanderbilt GCC study which reported that 80% of patients did not need additional appointments with a clinical geneticist (Hannig et al., 2014). Further exploration into the amount of downstream revenue generated by clinics where genetic counselors are practicing with minimal or no supervision by a geneticist is needed.

### 4.4 Study limitations

Missing or incomplete data related to patient demographics and referral information were the main limitation of the study and are known limitation of conducting a medical chart review (Sarkar & Seshadri, 2014). When there was missing or incomplete information within the electronic medical record, data were crossed referenced with other records. Even with this cross‐examination, many patients' wait times were unable to be recorded. Additionally, investigators still anticipate a referral bias from primary care providers due to this provider type being automatically listed as the default provider in the medical record.

### 4.5 Research recommendations/Future directions

The study outlines the most frequently seen patient indications for the UNMC GCC and evaluates the utilization of this clinic model. Clinical recommendations were analyzed for the GCC; however, the patient uptake of these recommendations remains unclear. Future research should explore uptake of specialist recommendations made in this clinic to better describe patient outcomes and potential downstream revenue generated by this clinic.

The chart review was completed for patients that were seen before March 16, 2020. After that date, the service delivery model for the GCC was transitioned from in‐person to telehealth. This shift in service delivery model allows future research to explore the impact telehealth may have on patient demographics, patient indication, clinical recommendations, and catchment area. Additional studies are planned to compare the variables evaluated in this study when patients were seen in person with a time frame where patients were only seen via telehealth. It is also possible that the telehealth model impacted patient uptake of this clinic model.

## 5 CONCLUSION

The results of this study can serve as a guide for genetic counselors, geneticists, or other providers looking to develop GCCs at their institution. By adapting the GCC model, healthcare systems can take action to decrease patient wait times for genetic services, alleviate geneticist workload, and facilitate 'top of scope' genetic counseling practice.

## AUTHOR CONTRIBUTIONS

Author Charles King confirms that he had full access to all the data in the study and takes responsibility for the integrity of the data and the accuracy of the data analysis. Charles King designed study goals and methods, performed data collection and analysis, wrote and revised the manuscript, and confirms he had access to all the data in the study and takes responsibility for the integrity of the data and accuracy of data analysis. Andrew Schmanski, Shelly Nielsen, Omar Rahman, and Kristen Fishler provided oversight and contributed to the study design, data interpretation, and review of the manuscript. All authors gave final approval of this version to be published and agreed to be accountable for all aspects of the work in ensuring that questions related to the accuracy or integrity of any part of the work are appropriately investigated and resolved.

## COMPLIANCE WITH ETHICAL STANDARDS

### Conflict of interest

Authors Charles King, Shelly Nielsen, Andrew Schmanski, Omar Abdul‐Rahman, and Kristen P. Fishler declare that they have no conflicts of interest.

### Human studies and informed consent

This study was approved by and conducted according to the ethical standards of the University of Nebraska Medical Center Institutional Review Board (IRB #: 452‐20‐EP). All applicable international, national, and/or institutional guidelines were followed. This study was approved by the IRB after expedited review and was granted an informed consent waiver.

### Animal studies

No non‐human animal studies were carried out by the authors for this article.

### Data sharing and data accessibility

The data that support the findings of this study are available upon reasonable request from the corresponding author. The data are not publicly available due to privacy or ethical restrictions.
